# Differential models of twin correlations in skew for body-mass index (BMI)

**DOI:** 10.1371/journal.pone.0194968

**Published:** 2018-03-28

**Authors:** Siny Tsang, Glen E. Duncan, Diana Dinescu, Eric Turkheimer

**Affiliations:** 1 Department of Epidemiology, Columbia University, New York, NY, United States of America; 2 Department of Nutrition & Exercise Physiology, Washington State University–Health Sciences, Spokane, WA, United States of America; 3 Kennedy Krieger Institute and Johns Hopkins School of Medicine, Baltimore, MD, United States of America; 4 Department of Psychology, University of Virginia, Charlottesville, VA, United States of America; University of Palermo, ITALY

## Abstract

**Background:**

Body Mass Index (BMI), like most human phenotypes, is substantially heritable. However, BMI is not normally distributed; the skew appears to be structural, and increases as a function of age. Moreover, twin correlations for BMI commonly violate the assumptions of the most common variety of the classical twin model, with the MZ twin correlation greater than twice the DZ correlation. This study aimed to decompose twin correlations for BMI using more general skew-t distributions.

**Methods:**

Same sex MZ and DZ twin pairs (*N* = 7,086) from the community-based Washington State Twin Registry were included. We used latent profile analysis (LPA) to decompose twin correlations for BMI into multiple mixture distributions. LPA was performed using the default normal mixture distribution and the skew-t mixture distribution. Similar analyses were performed for height as a comparison. Our analyses are then replicated in an independent dataset.

**Results:**

A two-class solution under the skew-t mixture distribution fits the BMI distribution for both genders. The first class consists of a relatively normally distributed, highly heritable BMI with a mean in the normal range. The second class is a positively skewed BMI in the overweight and obese range, with lower twin correlations. In contrast, height is normally distributed, highly heritable, and is well-fit by a single latent class. Results in the replication dataset were highly similar.

**Conclusions:**

Our findings suggest that two distinct processes underlie the skew of the BMI distribution. The contrast between height and weight is in accord with subjective psychological experience: both are under obvious genetic influence, but BMI is also subject to behavioral control, whereas height is not.

## Introduction

Body Mass Index (BMI) is one of the more intensively studied phenotypes in the genetic epidemiology literature. A 2012 meta-analysis identified 88 individual effect sizes from twin studies [1] and estimated a mean heritability of 0.73 in males and 0.76 in females, with only a small percentage originating in the shared environment and the remainder from the non-shared environment. Several characteristics of BMI suggest that there may be more to its heritability than meets the eye, however [2, 3]. First, the distribution of BMI has a strong positive skew [4], which appears to shift towards the upper end of the distribution as a function of age [5, 6]. Second, twin correlations for BMI commonly violate the assumptions of the standard version of the classical twin model, called the ACE model, which partitions the variability of the phenotype into components attributable to the additive effect of genes (A) and shared and non-shared environments (C and E respectively). An expectation of this simple model is that the correlation between DZ twins should be at least half the correlation between MZ twins. In an earlier meta-analysis that listed the individual MZ and DZ twin correlations [7], the MZ twin correlation was greater than twice the DZ correlation for 24 out of 64 reported effects (38%). These findings suggest that something other than the additive effects of individual genetic loci plus the independent effects of environmental factors is contributing to twin resemblance.

The primary goal of this study was to use latent profile analysis (LPA) to decompose the twin correlations and corresponding biometric components for BMI into multiple distributions using a large sample of twins. LPA estimates statistical models in multiple classes determined by the structure of the data. Our analysis differs from typical applications of LPA to twin studies in several ways. First, it has recently become possible to loosen the classical method of decomposing variables into normally distributed latent profiles (or classes); recent developments using the software Mplus allow dependent variables to be decomposed into more general skew-t distributions [4], making them much more flexible in the analysis of skewed outcomes. Although BMI is often log-transformed in statistical analyses [8, 9] to create a normal distributed phenotype, we contend that in transforming BMI important information about the scale and skewness of the distribution of body mass is lost.

Second, the usual strategy in LPA-based models of twin studies has been to estimate the classes on the individual phenotypes, and subsequently estimate the twin correlations for the estimated classes [10, 11]. Here, we estimate the classes at the pair level, thus including the twin correlations among the parameters being optimized by the LPA. Throughout, we compare our BMI results to those for height, a normally distributed trait that does not produce results at odds with the classical twin model. We hypothesize that the twin correlations for BMI can be decomposed into a relatively normally distributed component within the normal range of BMI, and a positively skewed component in the overweight and obese range of BMI. In contrast, we hypothesize that the twin correlations for height will consist of one normally distributed component.

## Methods

The study was approved by the IRB at the University of Virginia (SBS #2014036900).

### Subjects

This study included a sample of 7,086 (4,753 MZ; 2,300 DZ) twin pairs from the community-based Washington State Twin Registry within a cross-sectional study design. Twins include same-sex male and female twin pairs aged 18–97 years, reared together. Participants were recruited from Washington State driver’s license and identification card applications [12]. All twins completed an enrollment survey with questions related to childhood similarity to evaluate twin zygosity (MZ vs. DZ), a common twin registry practice with an accuracy of 95–98% compared to biological indicators [13, 14]. Twins were mailed an invitation letter and enrollment survey including questions related to height and weight. Data collected from completed questionnaires received between 2009 and 2015 were analyzed.

### Body mass index

The main outcome was BMI calculated from self-reported height and weight and expressed as *kg*/*m*^2^. These measures were collected from responses to the survey questions “What is your current height?” in feet and inches and “What is your current weight?” in pounds. In a sample of twins (*n* = 144 pairs) participating in an ongoing funded study [15], there was excellent agreement between mean self-reported and measured BMI (26.7 vs. 27.5 *kg*/*m*^2^, respectively; *r* = 0.97), suggesting our use of self-reported height and weight for BMI is an acceptable measure.

### Statistical analyses

Latent profile analyses (LPA) of BMI were estimated for the one, two, and three class models using the default normal distributions. As the distribution of BMI is positively skewed [4], non-normal mixture modeling with the skew-t distribution was also estimated for the one, two, and three class models. The skew-t distribution takes into account excessive skewness and kurtosis of the BMI distribution by including parameters for skew and degrees of freedom [16, 17].

We conducted the profile analysis on a simple model of the between and within pair variances of BMI in the twins. The models were fitted at the twin-pair level, with the latent classes estimated based on the within-pair and between-pair means and variances of the twin pairs. The between-pair means were constrained to be equal between MZ and DZ twin pairs but varied across the classes, whereas the within-pair means were fixed at zero, as is typical of two-level models. The between- and within-pair variances were allowed to vary between MZ and DZ twin pairs and across classes. The sum of the between- and within-pair variances (equal to the total phenotypic variance) were constrained to be equal between MZ and DZ twin pairs, but were allowed to vary across classes. Skew parameters for the between- and within-pair variances, and degrees of freedom for each class were also estimated in the skew-t mixture models. The ratio of the between pair variance to the phenotypic variance estimates the intraclass correlation for the twins. We refer to these models as intraclass correlation models.

As a comparison, similar mixture models were performed for height. Considering the differences in average BMI and height between men and women, the mixture models were fitted separately for each sex.

All mixture models were estimated using 100 random starting values and 20 final stage optimizations in order to replicate the best log-likelihood. The log-likelihood (LL), Bayesian information index (BIC; [18]) and entropy were reported for each latent class mixture model. The BIC imposes a penalty term to the LL for the number of model parameters, with a lower BIC value indicative of better model fit [19, 20]. Entropy is a measure of classification accuracy; a model with entropy closer to 1 suggests greater classification accuracy [21]. Three criteria were used to determine the best latent class solution: the lowest BIC value relative to the other models, a substantively meaningful model, and adequate group membership per latent class (at least 10% of the sample).

The probability that each twin pair belonged to each of the latent classes was estimated based on the data and the maximum likelihood parameter estimates associated with the mixture model. Twin pairs were assigned to membership in the latent class to which they had the highest likelihood of being a member. All mixture modeling and twin analyses were performed using Mplus version 7.4 [22].

## Results

### Descriptive statistics

**Fig 1:**
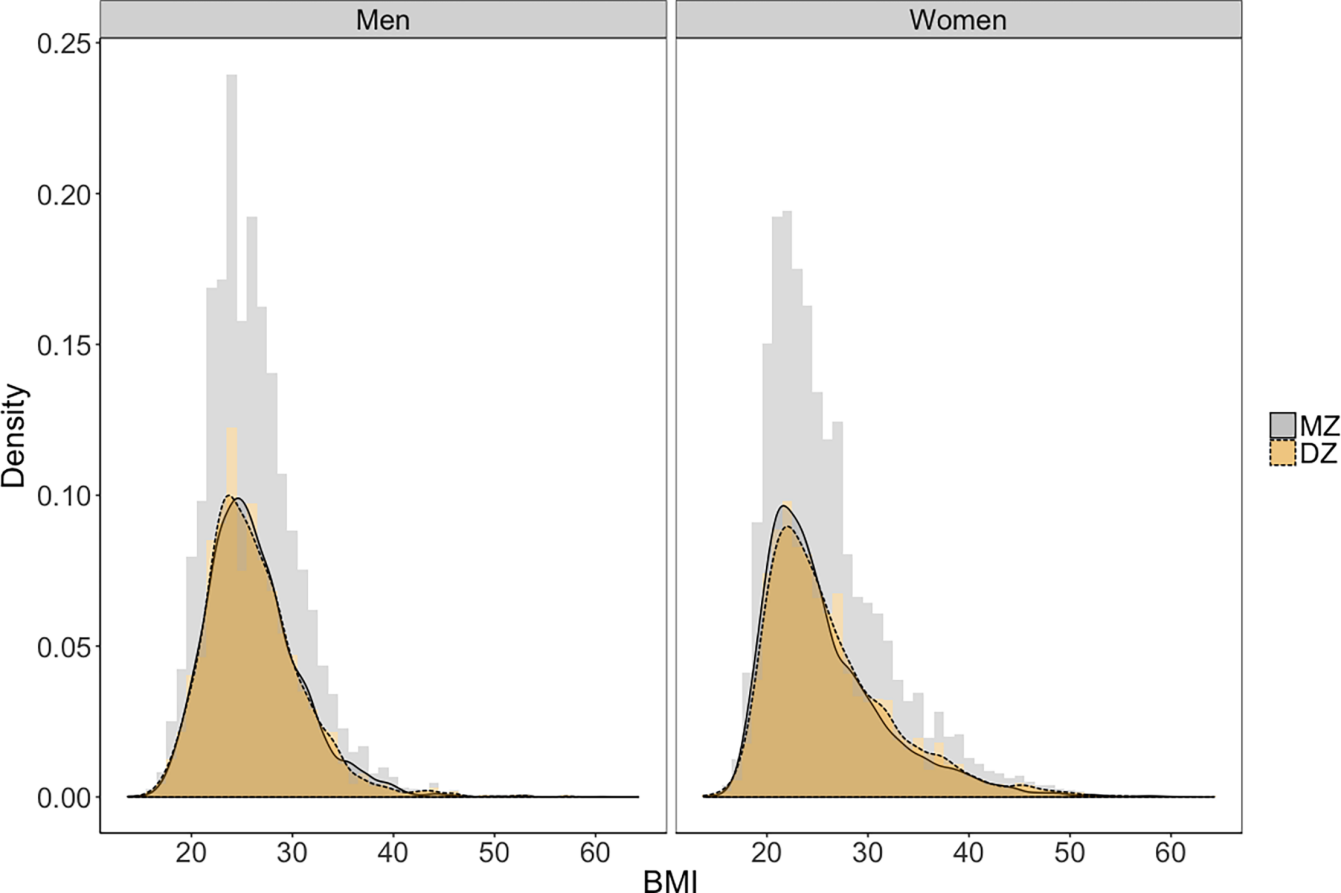
Distribution of BMI by gender.

**Fig 2:**
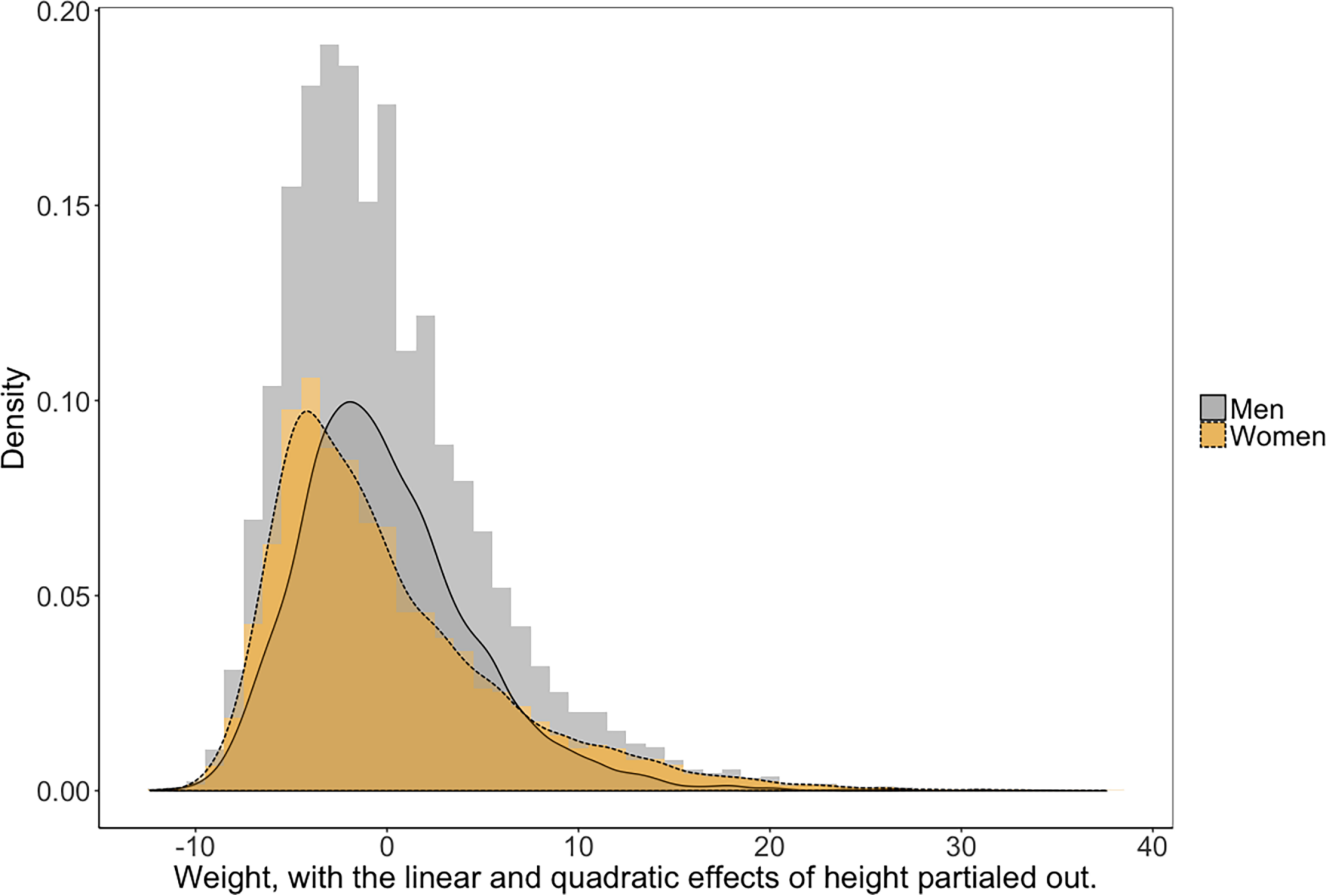
Distribution of weight, with the linear and quadratic effects of height partialed out, by gender.

**Fig 3:**
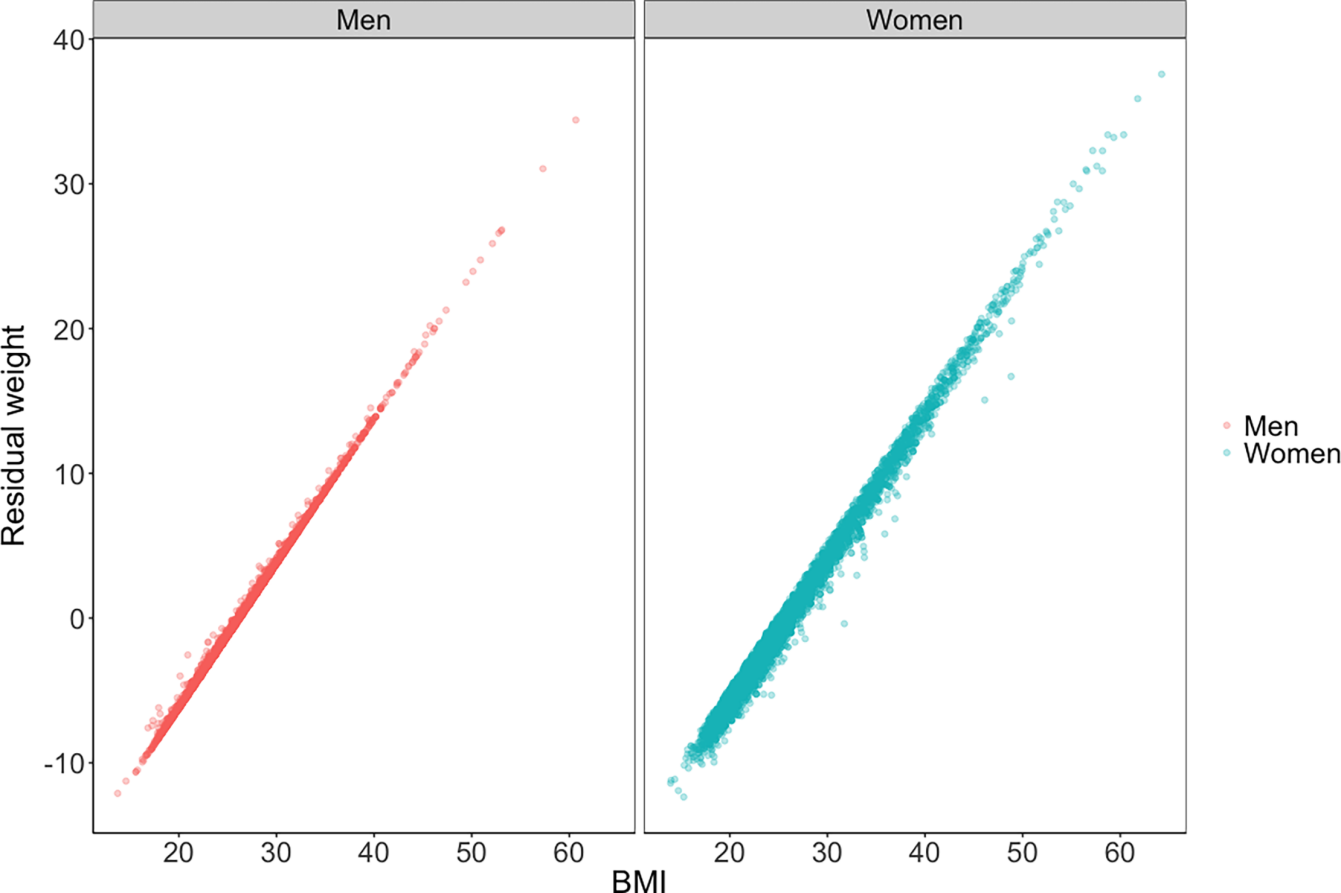
Association between BMI and weight, with the linear and quadratic effects of height partialed out, by gender.

Descriptive statistics and the distribution of BMI by gender and zygosity are presented in Table 1 and Fig 1. As expected, BMI is positively skewed in both men and women. We also compared BMI to the distribution of weight, with the linear and quadratic effects of height partialed out. This distribution was identical to the BMI distribution (*r* = 1 for both gender, Figs 2 and 3), suggesting that the skew of the BMI distribution is not a peculiarity of the way BMI is calculated, but is in fact, a structural property of human body size.

**Table 1:** Descriptive statistics of body-mass index (BMI).

		BMI				BMI residual			
Gender	Zygosity	*M*	Var	Skew	Kurt	*M*	Var	Skew	Kurt
Men	MZ	26	22	1.1	2.7	0.01	22	1.1	2.6
	DZ	26	23	1.2	3.5	-0.01	22	1.2	3.5
Women	MZ	26	37	1.5	3.0	-0.20	37	1.5	3.1
	DZ	26	40	1.3	2.2	0.43	39	1.4	2.2

*M* = mean. Var = variance. Skew = skewness. Kurt = kurtosis.

We began by estimating twin correlations and a classical twin (ACE) model for BMI in men and women. In men, the MZ twin correlation (*r*_*MZ*_) was 0.71 (*SE* = 0.02), the DZ twin correlation (*r*_*DZ*_) was 0.36 (*SE* = 0.04). In women, *r*_*MZ*_ was 0.73 (*SE* = 0.01), *r*_*DZ*_ was 0.44 (*SE* = 0.02). These correlations are consistent with the heritability coefficient [2(*r*_*MZ*_ – *r*_*DZ*_)] of 0.70 and 0.58 for men and women; a small proportion of the variance attributed to the shared family environment (2*r*_*DZ*_ – *r*_*MZ*_ = 0.01 and 0.15, for men and women) and non-shared environment and measurement error (1 - *r*_*MZ*_ = 0.29 and 0.27 for men and women). These findings are consistent with previous reports of twin studies of BMI [23].

### Latent profile analyses

#### BMI

The fit statistics of the mixture models under normal and skew-t distributions for BMI are presented in Table 2. The one-class skew *t* distribution mixture models fit better than the one-class normal distribution mixture models for both men and women (*BIC*_*skewt*_ = 30805 and *BIC*_*normal*_ = 31570 for men; *BIC*_*skewt*_ = 58163 and *BIC*_*normal*_ = 60924 for women), indicating that excessive skewness and kurtosis need to be taken into account when modeling the distribution of BMI. For both men and women, the two-class skew-t distribution mixture models had lower BICs than the one- and three-class skew-t mixture models, suggesting that the two-class solutions were better fit for both gender. The entropy value probabilities were 0.723 and 0.783 for men and women, respectively, suggesting adequate latent class separation (18). For men, the three-class skew-t distribution mixture model had zero individuals in one of the latent classes, suggesting that a third latent class was not needed. Similar results were found for women: one of the latent classes had less than 2% of the participants, suggesting that the three-class skew-t distribution mixture model was not a good fit.

**Table 2:** Fit statistics of the intraclass correlation mixture models under normal and skew-t distributions for BMI and height.

			Normal				Skew-t			
	Gender	Class	*df*	LL	BIC	Entropy	*df*	LL	BIC	Entropy
BMI	Men	1	5	-15765	31570	-	6	-15379	30805	-
		2	10	-15323	30725	0.752	12	-15270	30633	0.723
		3	15	-15256	30630	0.739	19	-15265	30678	0.799
	Women	1	5	-30441	60924	-	6	-29056	58163	-
		2	10	-28887	57859	0.82	12	-28727	57555	0.783
		3	15	-28637	57401	0.749	19	-28728	57616	0.82
Height^a^	Men	1	5	-12964	25967	-	7	-12660	25376	-
		2	10	-12596	25270	0.91	-	-	-	-
	Women	1	5	-22892	45827	-	7	-22318	44695	-
		2	10	-22223	44531	0.92	-	-	-	-

*df* = degrees of freedom. LL = log-likelihood. BIC = Bayesian information index. Entropy is not available for one-class models as there was no class separation.

^a^ The three-class solutions under the normal mixture distribution are omitted here as one latent class had zero observations for men, and two latent classes had less than 10% of the sample for women. Only the one-class solutions are presented for the skew-t mixture distribution as results showed only excessive kurtosis, but not excessive skewness, needed to be accounted for.

Table 3 shows the estimated parameters from the two-class skew-t mixture models. In both men and women, the solution consisted of one relatively non-skewed distribution, with mean BMI in the normal range (*M* ~ 22kg/*m*^2^ for both men and women), and a skewed distribution, with mean BMI in the overweight range (*M* ~ 29kg/*m*^2^ for both men and women). We subsequently labeled the latent profile with mean BMI in the normal range as the "normal" class, and the other one the "overweight" class.

**Table 3:** Descriptive statistics and twin correlations of BMI and height for the intraclass correlation mixture models.

	Gender	Profile	Zyg	N (%)	*M*	*SD*	Skew	Kurt	*r* (95% CI)
**BMI**	Men	Normal	MZ	690 (40.9%)	22.7	2.0	-0.5	-0.4	0.79 [0.77, 0.82]
			DZ	325 (38.7%)	22.6	2.0	-0.4	-0.2	0.28 [0.18, 0.38]
		Overweight	MZ	995 (59.1%)	28.5	4.5	1.0	3.5	0.5 [0.45, 0.54]
			DZ	514 (61.3%)	28.4	4.6	1.2	4.2	0.01 [-0.07, 0.1]
	Women	Normal	MZ	1595 (52%)	21.9	2.1	0.0	-0.7	0.71 [0.69, 0.73]
			DZ	649 (44.4%)	22.0	2.1	-0.1	-0.6	0.29 [0.22, 0.36]
		Overweight	MZ	1470 (48%)	29.8	6.3	1.1	2.0	0.5 [0.46, 0.54]
			DZ	812 (55.6%)	29.6	6.5	0.9	1.3	0.14 [0.07, 0.2]
**Height**	Men		MZ	1649 (100%)	5.9	0.2	-0.1	0.3	0.85 [0.84, 0.86]
			DZ	749 (100%)	5.9	0.2	0.2	1.6	0.5 [0.44, 0.55]
	Women		MZ	2930 (100%)	5.4	0.2	0.1	1.6	0.82 [0.81, 0.84]
			DZ	1290 (100%)	5.4	0.2	0.3	3.0	0.49 [0.45, 0.53]

Zyg = zygosity. *M* = mean. *SD* = standard deviation. Skew = skewness. Kurt = kurtosis. *r* = *r*_*MZ*_ for MZ twin pairs; *r*_*DZ*_ for DZ twin pairs. 95% CI = 95% confidence interval.

**Fig 4:**
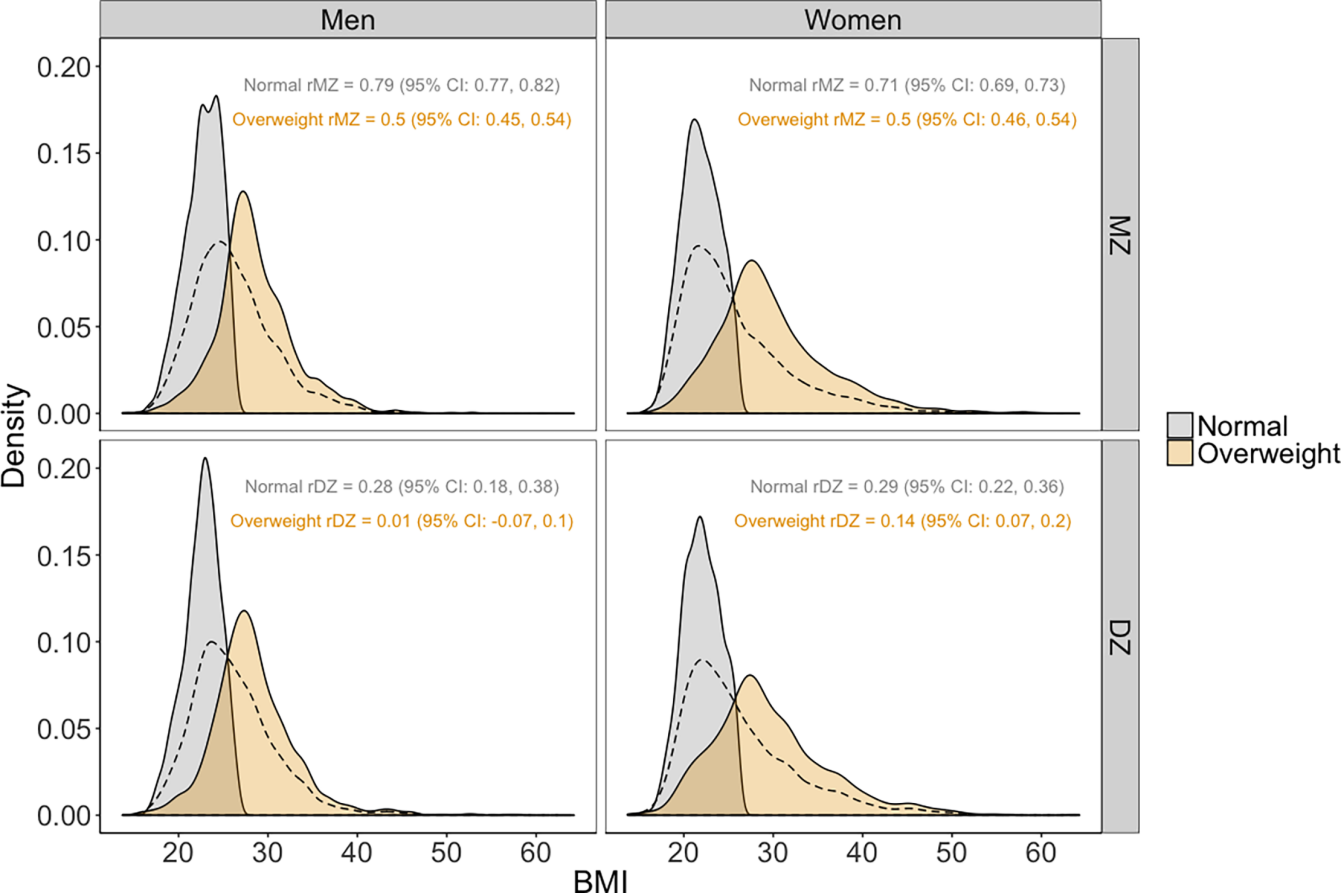
BMI density of the two latent classes by gender and zygosity. *Note*. The densities of the single-class BMI are illustrated in dashed lines.

Table 3 presents the number and proportion of twin pairs in each latent profile by gender and zygosity. Fig 4 illustrates the distributions of BMI in the normal (in grey) and overweight (in tan) latent classes. Consistent across gender and zygosity, the normal profile shows a relatively normal distribution, with a mean BMI of between 21 and 22 units, and a small variance. All twins in the normal class have BMI ≤ 30. The overweight class shows a positively skewed distribution, with a mean BMI of about 29 units and a much larger variance.

**Fig 5:**
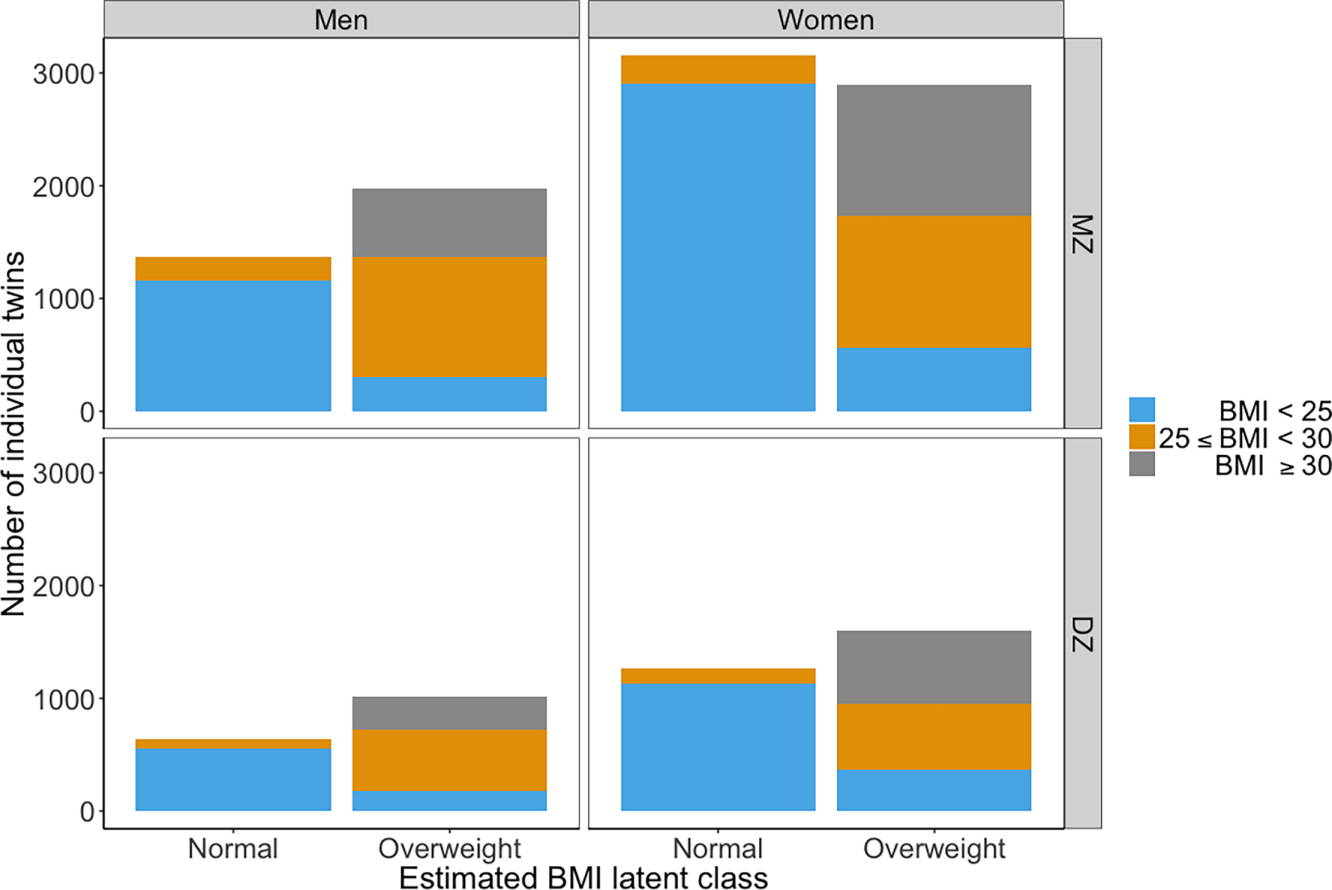
Distribution of participants in normal and overweight class, stratified by BMI.

The number of individual twins in each latent class stratified by their measured BMI is presented in Fig 5. For both men and women, the majority of twins in the normal class have BMIs within the normal range (BMI < 25), and only a very small proportion have BMIs in the overweight range (25 ≤ BMI < 30). In the overweight class, the proportion of twins with normal BMI was relatively small, whereas most twins have BMIs in the overweight or obese range (BMI ≥ 30).

**Fig 6:**
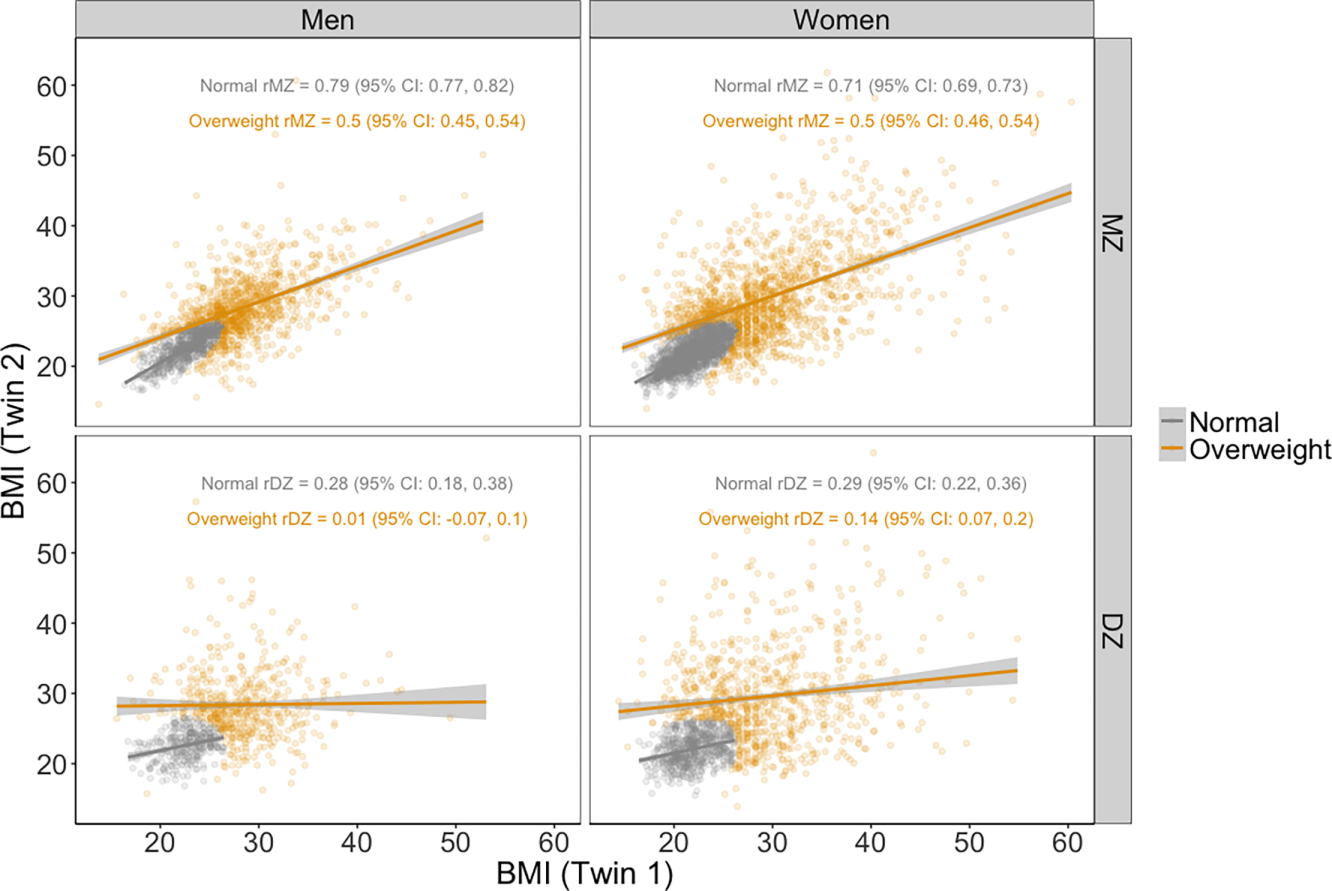
Associations of BMI between twin pairs of the two latent classes by gender and zygosity.

The twin correlations in the normal class (*r*_*MZ*_ = 0.79 and *r*_*DZ*_ = 0.28 for men; *r*_*MZ*_ = 0.71 and *r*_*DZ*_ = 0.29 for women) are indicative of high heritability, no effect of family environment, a relatively small effect of non-shared environment, and a modest violation of the ACE model. In contrast, the smaller twin correlations in the overweight class (*r*_*MZ*_ = 0.5 and *r*_*DZ*_ = 0.01 for men; *r*_*MZ*_ = 0.5 and *r*_*DZ*_ = 0.14 for women) suggest a large non-shared environmental effect and a severe violation of the ACE model. These twin correlations are illustrated in Fig 6. MZ twin correlations are higher than DZ twin correlations in both classes for men and women; correlations are higher in the normal class compared to the overweight class for both men and women, and for MZ and DZ twins.

To explore the extent to which the normal/overweight class distribution was associated with age, we computed the Pearson correlations between age and the estimated class probabilities of being in the overweight class. The estimated class probability was used to take into account measurement error in the estimation of the most likely latent class membership. Twins in the overweight class were, on average, older (mean age = 44 to 48 years old) than those in the normal class (mean age = 34 to 40 years old).

#### Height

The fit statistics of the mixture models under normal and skew-t distributions for height are presented in Table 2. Among the one-class models, the skew-t distribution mixture models had lower BICs than the normal mixture models. The degree of freedom parameters (*df* = 5.80, *SE* = 0.51, *p* < .0001 for men; *df* = 6.27, *SE* = 0.47, *p* < .0001 for women) deviated from that of the normal distribution (*df* ≥ 30), whereas the skew parameters were non-significant (skew = -0.07, *SE* = 0.16, *p* = 0.687 for men; skew = 0.18, *SE* = 0.14, *p* = 0.188 for women). These results suggested the need to account for excessive kurtosis but not excessive skewness for height. Subsequent multi-class mixture models were only estimated using normal distributions.

**Fig 7:**
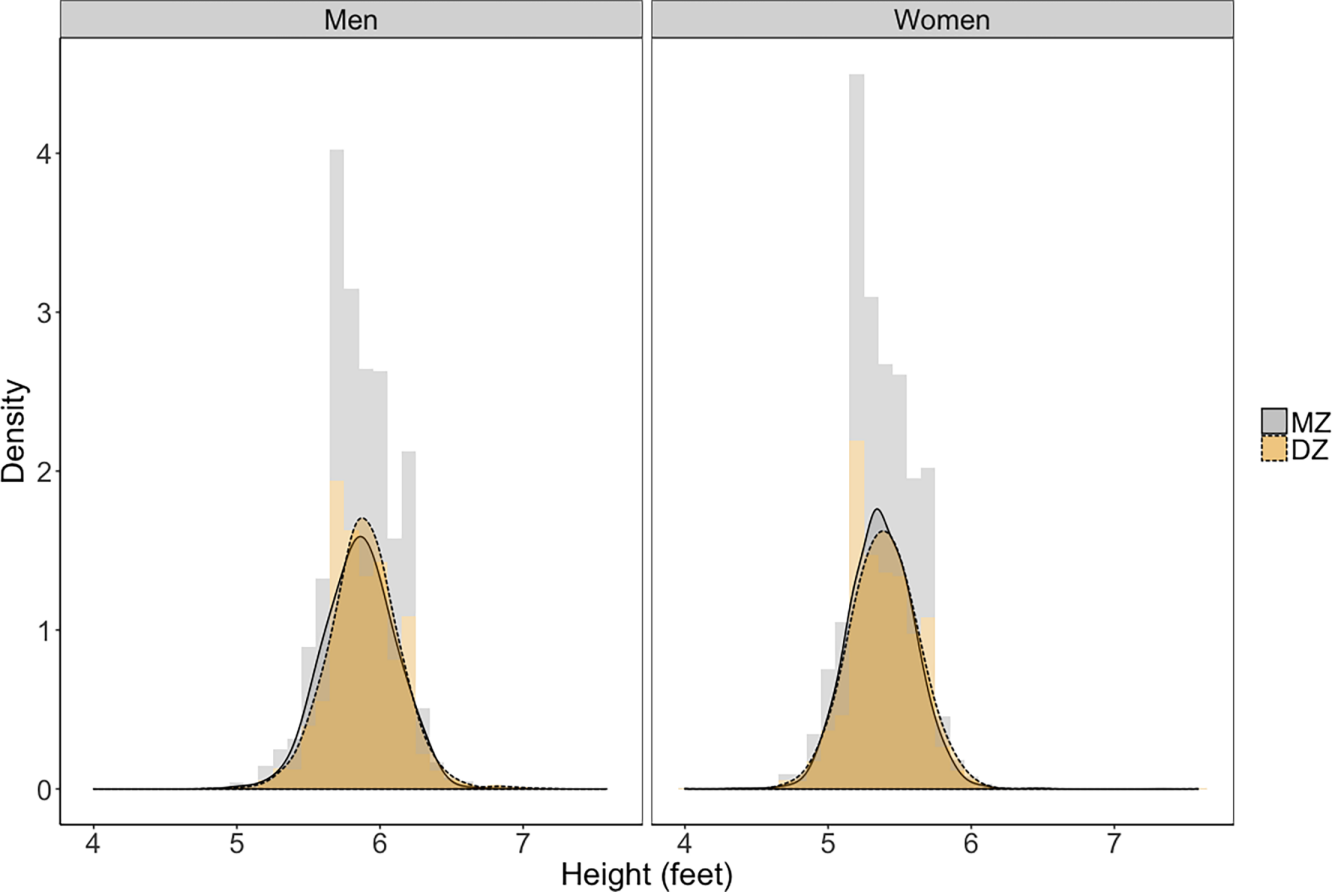
Distribution of height (in feet) between twin pairs by gender and zygosity.

For both men and women, although the two-class mixture models showed improvements in BIC over the one-class mixture model, more than 90% of the participants were estimated to belong to one class (94% for men and 95% for women), suggesting that the one-class solution was a sufficient fit to the current data for both men and women. Descriptive statistics of height by sex and zygosity are presented in Table 3. The distributions of height are almost identical between MZ and DZ twin pairs, with men being slightly taller than women (Fig 7). It should also be noted that the distribution of height is relatively normal for both men and women.

**Fig 8:**
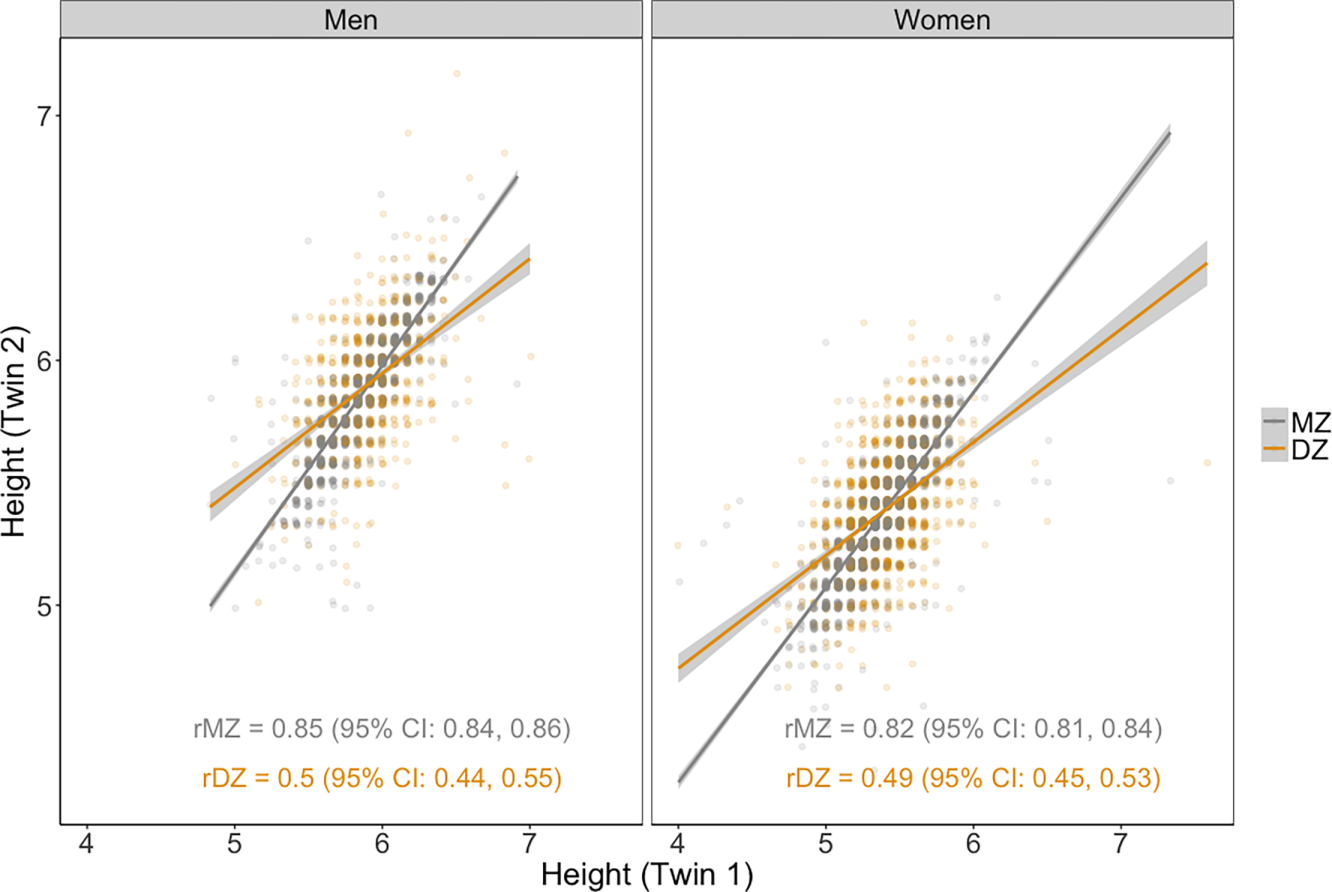
Associations of height (in feet) between twin pairs by gender and zygosity.

The twin correlations of height by sex and zygosity are also illustrated in Fig 8. The height of MZ twin pairs, regardless of sex, are highly correlated with each other, whereas moderate correlations were observed between DZ twin pairs. These results suggest high heritability of height, with very small family environment effects.

## Replication

We applied the same statistical analyses to decompose the twin correlations and corresponding biometric components for BMI into multiple distributions in another large independent cohort of twins for a replication study. This cohort included 13,553 (5,965 MZ, 7,588 DZ) twin pairs from the National Academy of Sciences-National Research Council Twin Registry (NAS-NRC Twin Registry [24]). Twins were same-sex male twin pairs, average aged 45 (*SD* = 3.9, range = 40–56) years at the time height and weight data was collected.

The distribution of BMI is positively skewed for MZ (*M* = 21.9, variance = 6.0, skew = 0.98, kurtosis = 2.21) and DZ (*M* = 22.1, variance = 6.3, skew = 1.09, kurtosis = 2.79) twins. With *r*_*MZ*_ = 0.80 and *r*_*DZ*_ = 0.42, twin correlation is consistent with the heritability of 0.76 for BMI; a small proportion of the variance attributed to the shared family environment (2*r*_*DZ*_ − *r*_*MZ*_ = 0.04) and non-shared environment and measurement error (1 - *r*_*MZ*_ = 0.20).

Results showed that the two-class skew-t distribution mixture model was the best fit for the intraclass correlation model (S1 Table). The two-class skew-t solution consisted of a normal BMI class (*M* ~ 21kg/*m*^2^) that is relatively normally distributed, and an overweight class (*M* ~ 24kg/*m*^2^) that is positively skewed (S2 Table and S1 Fig). The twin correlations in the normal class (*r*_*MZ*_ = 0.81 and *r*_*DZ*_ = 0.24) are indicative of high heritability, whereas those in the overweight class (*r*_*MZ*_ = 0.56 and *r*_*DZ*_ = -0.05) suggest a large non-shared environmental effect and a severe violation of the classical twin model (S2 Fig). Twins in the overweight class were, on average, older than those in the normal class (*r* = 0.12 and 0.16, for MZ and DZ twins, respectively; all *p*s < .001).

## Discussion

The current study presents a unique investigation of the skewed nature of BMI using latent profile analyses in a large sample of twin pairs. The BMI latent classes were estimated by including the twin correlations in the optimization process; twin pairs were therefore assigned to be in the same latent class. Our use of the skew-t distribution in the estimation accounts for the excessive skewness and kurtosis of the skewed distribution of BMI (4, 13, 14). The findings show that the skewness of the BMI distribution can be decomposed into a relatively normally distributed component within the normal BMI range, and a positively skewed component in the overweight and obese range of BMI, with the former more highly correlated in twin pairs than the latter. Twin correlations in the first class conformed to the assumptions of the classical twin model; those in the second class did not. Similar results were obtained when the analyses were replicated using another large sample of twin pairs.

Our analyses suggest that two distinct processes underlie the skew of the BMI distribution and the tendency for twin correlations for BMI to violate the assumptions of the classical twin model. The first process is relatively normally distributed, in the normal range of BMI, and highly heritable. The second process is positively skewed, in the overweight and obese range of BMI, with lower twin correlations, especially for DZ twins. The near-zero DZ twin correlations represent a severe violation of the assumptions of the classical ACE twin model, and for that matter any genetically based twin model, none of which predict correlations of zero in DZ twins. In contrast to BMI, height is normally distributed, highly heritable, does not violate the classical twin model, and is well-fit by a single latent class. This contrast between height and weight is in accord with human psychological and physiological experience of height and BMI: both are under obvious genetic influence, but BMI is also subject to individual behavioral and environmental control, whereas height is not [25].

Our finding that twins in the overweight class are more likely to be older than those in the normal class is consistent with the positive shift in BMI with age in other samples [5, 6]. It is possible that these changes in the BMI distribution over age may be largely due to the positively skewed distribution of BMI, reflecting increased environmental contribution to the change in BMI with age. That is, BMI in the normal range may be largely inherited, whereas BMI above the normal range accrues during the lifespan according to individual level processes that are less correlated in twin pairs. A meta-analysis of 40 twin cohorts showed that the heritability of BMI decreases with age in both men and women [26], suggesting that the gain in BMI, which is reflected in the skewness of the BMI distribution, may be less heritable than the BMI distribution within the normal range. Nonetheless, this hypothesis with respect to the two distinct processes of BMI needs to be tested with data in which repeated measures BMI are collected across ages.

A few limitations of this study should be noted. First, height, weight and zygosity were all self-reported. Second, participants were primarily Caucasians, which limits generalizability of our findings to other race/ethnic groups. Future research should replicate our findings among populations of different race/ethnic composition. Third, the cross-sectional nature of our data limits our ability to examine potential differential causal relationships between BMI, genetic, behavioral, and environmental factors. Future research should make use of longitudinal studies to examine such associations, and investigate how components of BMI change or stabilize across the lifespan.

Deeper understanding of the nature of these weight classes will require more information at the genetic, environmental, and phenotypic level. It would be interesting to know, for example, whether either candidate genes or polygenic risk scores are more correlated with one distribution than the other. Similarly, one could investigate how dietary or activity behaviors, or food and activity environments, differ between the classes, or how the classes change or stabilize across the lifespan.

We have explored statistical models of twin development that emphasize reciprocal effects of differences in phenotype and individual behavior, demonstrating that such processes have the result of systematically depressing DZ twin correlations [27]. To date these models have mostly been applied to cognitive development, but they would appear to be broadly applicable to BMI as well. It should also be noted that positively skewed distributions are characteristic of a wide range of human phenotypes, like alcohol consumption [28] and mood [29], that comprise both normal and disrupted behavior. Many of these same human phenotypes frequently violate the classical twin model in the same way as BMI, with MZ twin similarity more than twice that of DZ twins [30, 31]. We hypothesize that many of these phenotypes consist of a normally distributed portion in the normal range under strong genetic control, and a skewed portion with a mean in the pathological range, representing environmental or reciprocal processes at the individual level.

## Supporting information

S1 Table Fit statistics of the mixture models under normal and skew-t distributions for BMI (NAS-NRC Twin Registry sample). (DOCX)

S2 Table Descriptive statistics and twin correlations of BMI for the intraclass correlation mixture models (NAS-NRC Twin Registry sample). (DOCX)

S1 Fig BMI density of the two latent classes by zygosity (NAS-NRC Twin Registry sample). (PDF)

S2 Fig Associations of BMI between twin pairs of the two latent classes by zygosity (NAS-NRC Twin Registry sample). (PDF)
