# Supplementary material for: Differential models of twin correlations in skew for body-mass index (BMI)
Source: PLoS One. 2018 Mar 28;13(3):e0194968. doi: 10.1371/journal.pone.0194968 (PMC5874062; doi:10.1371/journal.pone.0194968)
Supplement: S1 Table — (DOCX) [file pone.0194968.s001.docx]

**S1 Table. Fit statistics of the mixture models under normal and skew-t distributions for BMI (NAS-NRC Twin Registry sample).**

|  |  | **Normal** | | | | **Skew-t** | | | |
| --- | --- | --- | --- | --- | --- | --- | --- | --- | --- |
|  | Class | *df* | LL | BIC | Entropy | *df* | LL | BIC | Entropy |
| BMI | 1 | 5 | -68521 | 137089 | - | 7 | -15379 | 133771 | - |
|  | 2 | 9 | -66640 | 133365 | 0.79 | 13 | -15270 | 133122 | .70 |
|  | 3 | 14 | -66438 | 133009 | 0.71 | 20 | -15265 | 133188 | .77 |

*df* = degrees of freedom. LL = log-likelihood. BIC = Bayesian information index. Entropy is not available for one-class models as there was no class separation.
