# Supplementary material for: Differential models of twin correlations in skew for body-mass index (BMI)
Source: PLoS One. 2018 Mar 28;13(3):e0194968. doi: 10.1371/journal.pone.0194968 (PMC5874062; doi:10.1371/journal.pone.0194968)
Supplement: S2 Table — (DOCX) [file pone.0194968.s002.docx]

**S2 Table. Descriptive statistics and twin correlations of BMI for the intraclass correlation mixture models (NAS-NRC Twin Registry sample).**

|  | **Profile** | **Zyg** | **N (%)** | ***M*** | ***SD*** | **Skew** | **Kurt** | $\boldsymbol{r}$**(95% CI)** |
| --- | --- | --- | --- | --- | --- | --- | --- | --- |
| **BMI** | Normal | MZ | 3713 (62.2%) | 20.6 | 1.4 | -0.3 | -0.4 | 0.81 [0.80, 0.82] |
|  |  | DZ | 4367 (57.6%) | 20.7 | 1.4 | -0.3 | -0.3 | 0.24 [0.22, 0.27] |
|  | Overweight | MZ | 2252 (37.8%) | 24.0 | 2.4 | 0.7 | 2.9 | 0.56 [0.53, 0.58] |
|  |  | DZ | 3221 (42.4%) | 23.9 | 2.5 | 0.9 | 2.9 | -0.05 [-0.08, -0.01] |

Zyg = zygosity. *M* = mean. *SD* = standard deviation. Skew = skewness. Kurt = kurtosis. *r* = $r_{MZ}$ for MZ twin pairs; $r_{DZ}$ for DZ twin pairs. 95% CI = 95% confidence interval.
