# Supplementary material for: Differential models of twin correlations in skew for body-mass index (BMI)
Source: PLoS One. 2018 Mar 28;13(3):e0194968. doi: 10.1371/journal.pone.0194968 (PMC5874062; doi:10.1371/journal.pone.0194968)
Supplement: S1 Fig — (PDF) [file pone.0194968.s003.pdf]

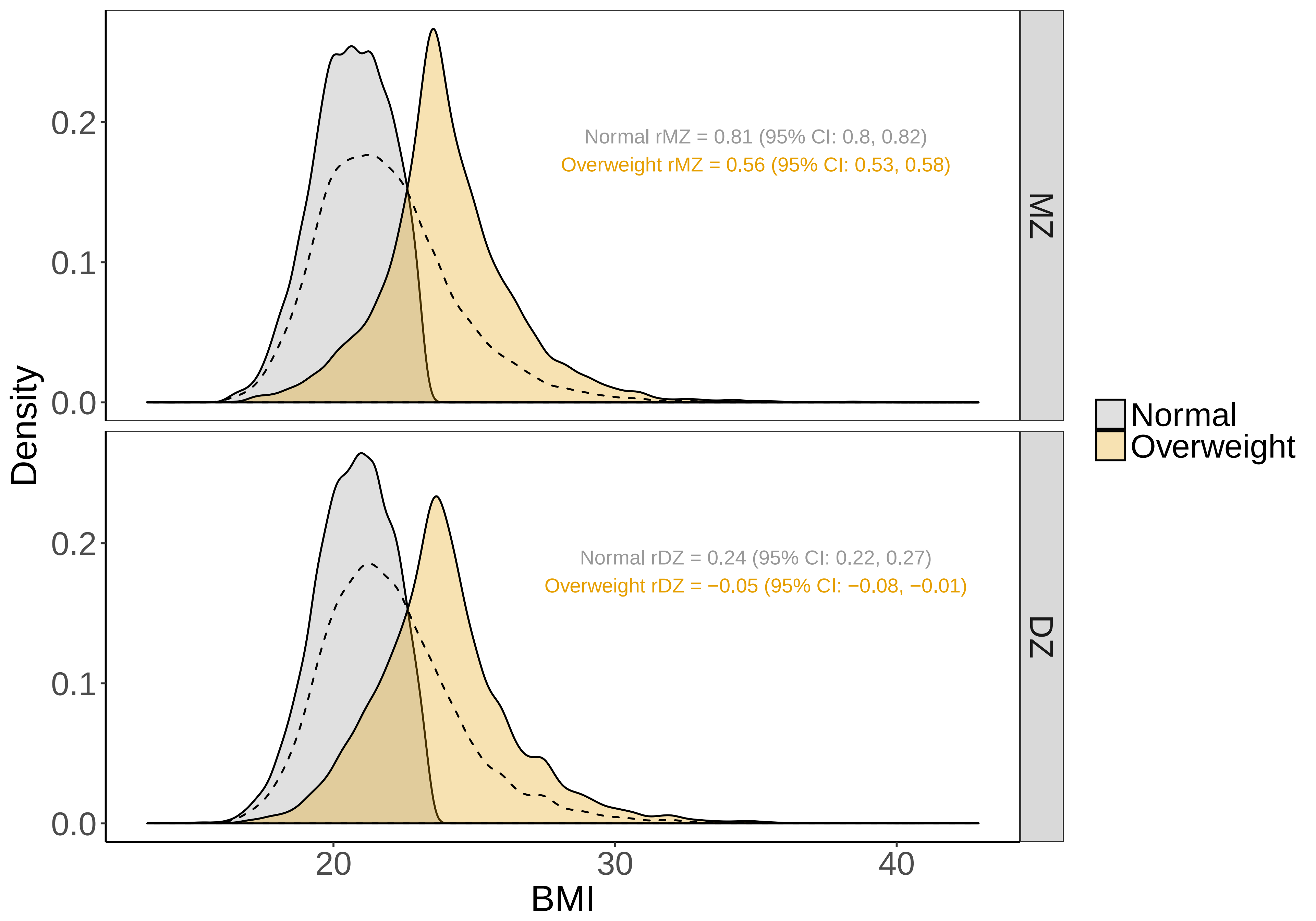
Normal rMZ = 0.81 (95% CI: 0.8, 0.82)
Overweight rMZ = 0.56 (95% CI: 0.53, 0.58)
MZ
Normal rDZ = 0.24 (95% CI: 0.22, 0.27)
Overweight rDZ = −0.05 (95% CI: −0.08, −0.01)
DZ
Density
0.0
0.1
0.2
20
30
40
BMI
Normal
Overweight
