# Supplementary figures and images for: Differential models of twin correlations in skew for body-mass index (BMI)
Source: PLoS One. 2018 Mar 28;13(3):e0194968. doi: 10.1371/journal.pone.0194968 (PMC5874062; doi:10.1371/journal.pone.0194968)

BMI (Twin 2)

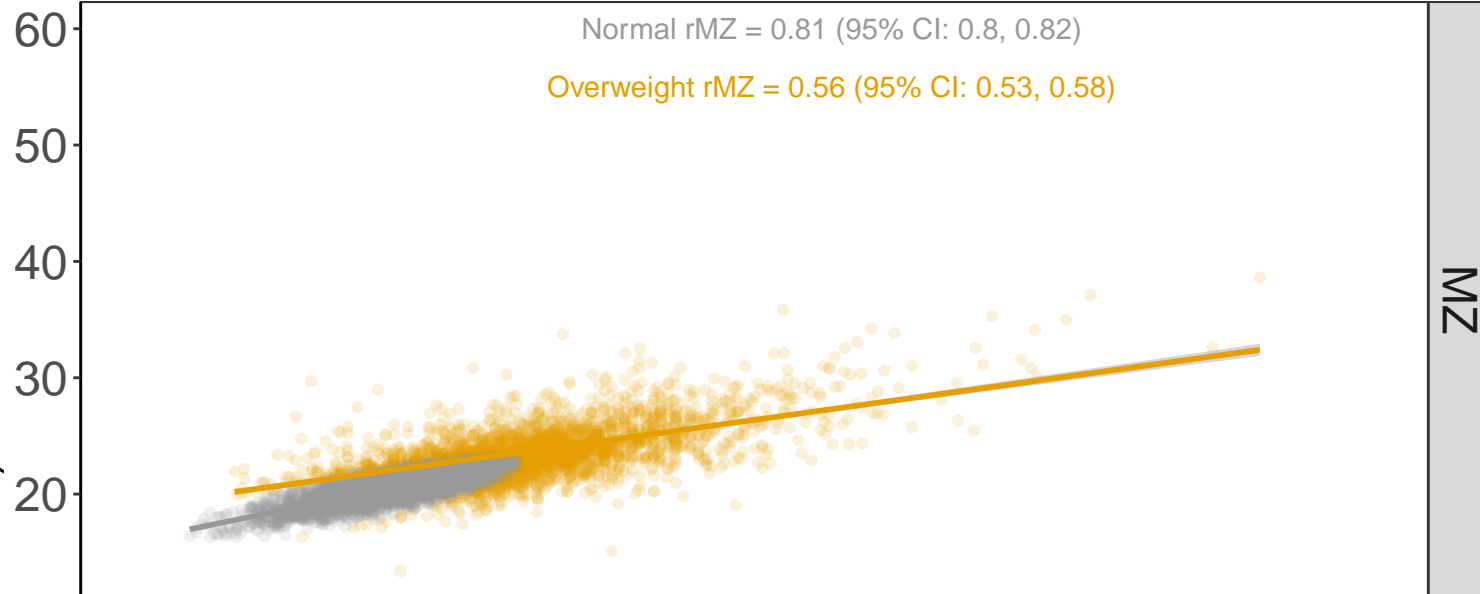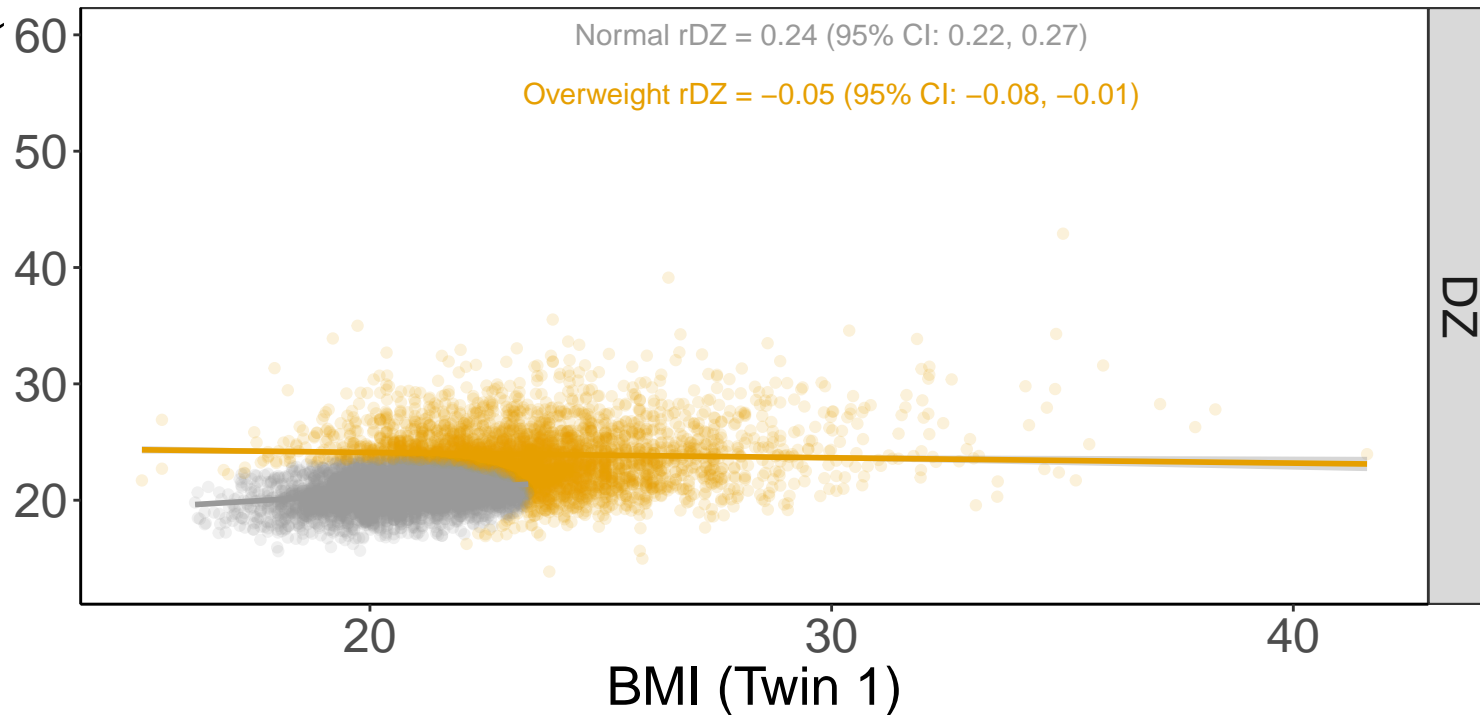

Normal

Overweight

Supplement: S2 Fig — (PDF) [file pone.0194968.s004.pdf]
